# Co-Evolution of Predator-Prey Ecosystems by Reinforcement Learning Agents

**DOI:** 10.3390/e23040461

**Published:** 2021-04-13

**Authors:** Jeongho Park, Juwon Lee, Taehwan Kim, Inkyung Ahn, Jooyoung Park

**Affiliations:** 1 Department of Control and Instrumentation Engineering, Korea University, 2511 Sejong-ro, Sejong-City 30019, Korea; seanpark0107@korea.ac.kr (J.P.); saero94j@korea.ac.kr (J.L.); kteaw0110@korea.ac.kr (T.K.); 2 Department of Mathematics, College of Science and Technology, Korea University, 2511 Sejong-ro, Sejong-City 30019, Korea; ahnik@korea.ac.kr

**Keywords:** predator and prey, population, co-evolution, ecosystem, reinforcement learning

## Abstract

The problem of finding adequate population models in ecology is important for understanding essential aspects of their dynamic nature. Since analyzing and accurately predicting the intelligent adaptation of multiple species is difficult due to their complex interactions, the study of population dynamics still remains a challenging task in computational biology. In this paper, we use a modern deep reinforcement learning (RL) approach to explore a new avenue for understanding predator-prey ecosystems. Recently, reinforcement learning methods have achieved impressive results in areas, such as games and robotics. RL agents generally focus on building strategies for taking actions in an environment in order to maximize their expected returns. Here we frame the co-evolution of predators and preys in an ecosystem as allowing agents to learn and evolve toward better ones in a manner appropriate for multi-agent reinforcement learning. Recent significant advancements in reinforcement learning allow for new perspectives on these types of ecological issues. Our simulation results show that throughout the scenarios with RL agents, predators can achieve a reasonable level of sustainability, along with their preys.

## 1. Introduction

The problem of addressing predator-prey interactions is an important field in ecology, and finding a reasonable population model for a predator-prey ecosystem is particularly important for understanding its dynamic features. Many researchers have studied population models with evolutionary dispersal perspectives, such as dispersal depending on other species [1,2,3,4] and starvation-driven diffusion depending on resources [5,6,7,8,9,10]. It is known that both the interaction between different species and the response of a species to its environment are necessary to develop a more realistic dispersal model for biological species [11,12]. For decades, researchers have developed dispersal theory based on the surrounding environment as an influential element [12], and the environment affecting a particular species includes elements, such as other interacting species. Because various species usually migrate to a region to find a more favorable habitat that provides sufficient food and/or better conditions for survival, an understanding of dispersal strategy is critically important to the study of species evolution. For a general explanation of discrete and continuous models on dispersal evolution, we refer the reader to References [12,13,14,15,16] and references therein. Many observations in nature demonstrate that the emergence of a predator or prey can induce the directed movement of a species; thus, researchers have proposed and investigated several mathematical models along these lines [17,18,19,20,21]. Despite the modeling and/or predictive capacity inherent within these mathematical approaches, the study of population dynamics still remains a challenging task in computational biology because analyzing and accurately predicting the intelligent adaptation of interacting species is difficult, and it is often desirable to employ an agent-based perspective to shed more light on the topic.

The agent-based approach (e.g., References [22,23]) is based on mutually interacting agents via prescribed rules in a simulated environment, and can efficiently and conveniently describe individual and mutual behaviors together. Contrary to the traditional tools of population dynamics modeling, the agent-based approach does not resort to directly modeling equilibrium through mathematics. Nevertheless, Monte Carlo simulation combined with the agent-based approach in two-dimensional space can often reveal more diverse and detailed spatiotemporal patterns arising in the domains under consideration. When the state transition rules and rewards are not stipulated in advance, as is typical in domains where reinforcement learning is applied, training is realized by means of interactions with the environment, which includes the responses of other agents, which enables learning agents to discover and evolve toward a better policy. Recently, reinforcement learning (RL) [24] has achieved impressive advances in areas, such as games [25,26]. RL agents generally focus on building strategies for taking actions in an environment in order to maximize expected rewards. Here we frame the co-evolution of predators and prey in an ecosystem as allowing agents to learn and evolve toward better ones in a manner appropriate for multi-agent learning. This leads us to new perspectives on the problem at hand, thanks to recent significant advancements in reinforcement learning. In this paper, we use a multi-agent version of reinforcement learning, which is often called MARL (multi-agent reinforcement learning) to understand and model the co-evolution process of predator-prey ecosystems. In general, multi-agent reinforcement learning is known to be much more challenging than single-agent cases, because agents in an environment need to learn together.

Many recent works have undertaken related approaches. As an important MARL application domain, one may consider games, such as Go and StarCraft. In this domain, MARL brings about breakthroughs exhibiting superhuman performance for complex environments (e.g., Atari, Go, chess, shogi, and StarCraft) [27,28]. Visually complex and challenging tasks were considered and addressed with great success by means of the MuZero algorithm [27], based on combining a tree-based search with a trained model, and utilizing self-play for each board game domain. StarCraft2 is considered by AlphaStar [28], which utilizes the strategies of multi-agent reinforcement learning and imitation learning. Similar to Muzero, AlphaStar also conducts self-play for training, and despite the game’s complexity, it turns out that AlphaStar reached the Grandmaster level for all races (Terran, Zerg, Protoss). Hahn et al. [29] considered swarms consisting of multi-agent individuals. For these individuals’ objectives, a multi-agent reinforcement learning (MARL) method based on Deep Q-Networks (DQN) is utilized to execute a foraging task. Ritz et al. [30] applied reinforcement learning to train a predator in a single predator and a multi prey system, in which a predator evolves by means of RL and interacts with preys, which are non-RL agents. In their works, a version of DQN was utilized for the RL framework with long-term reward discounting and stacked observations. Phan et al. [31] also considered a MARL method for a multi-agent system, in which they proposed a novel approach called Stable Emergent Policy (STEP) approximation. The STEP approximation method is trained by means of a decentralized planning approach, in which a simulator is used for executing the planning. After the decentralized planning procedure is over, the trained policy is reintegrated into the method. Hahn et al. [32] proposed Swarm Emergent Learning Fish (SELFish), a reinforcement learning approach to evolve prey animals based on predation. For the multi-agent concept, every individual agent optimizes its own behavior without any centralization or integration. This leads to an emergent flocking behavior. Gabor et al. [33] proposed a hybrid adversarial learner, in which one hybrid component is a reinforcement learning mechanism for problem solving, and the other components is an evolutionary algorithm for finding instances of problems. While this method was simulated with the problem scenario of a smart factory, it is almost identical to the predator-prey problem, since the smart factory problem is defined in a grid environment, and the goal of the agent is find a particular workstation. Hüttenrauch et al. [34] proposed a new RL state representation for swarm systems, and utilized ad-hoc feature spaces of the mean embedding based on histograms and radial basis functions, along with communication protocols. In Reference [35], Blasius et al. applied power analysis and bivariate wavelet analysis to quantify some statistical associations among the dynamics of predator and prey densities. The problem of formalizing the co-evolution of predators and preys as RL was recently introduced in Wang et al. [36], who claim that predators’ RL ability contributed to the stability of an ecosystem and helped predators attain more reasonable behavior patterns of coexistence with their prey; the RL effect of prey on its own population was not as successful as that of predators, and increased the risk of extinction of the predators. Their subsequent work [37] adopted neural networks and presented a similar RL-based evolution mechanism for predator-prey ecosystems, with discretized features for states. The methodologies used in these works are somewhat similar in essence to those of the present paper, but there are some distinctive differences in the final results, which are to be detailed in the discussion section below. Finally, for a comprehensive survey of recent progress in multi-agent reinforcement learning, the reader is referred to, e.g., Reference [38].

In this paper, we utilize the perspective of recent multi-agent reinforcement learning (MARL) approaches, and present a MARL-based description of co-evolution mechanisms in predator-prey ecosystems, in which agents shows biologically plausible approximation of their co-evolution over multiple generations in nature. Specifically, we present a simple procedure for deriving co-evolution of agents with imperfect observations in predator-prey ecosystems by directly optimizing for equilibrium policies via reinforcement learning. We empirically show the policies this procedure yields and show that they demonstrate sound equilibrium properties. In addition, we empirically show that some dynamic properties of real predator-prey systems can be replicated with simulations.

The remaining sections of the paper are organized as follows: In Section 2, we describe the environment for simulations, along with learning agents. We explain the environment rules, and observations and rewards that characterize the learning agents. In addition, we introduce our multi-agent RL approach for agents’ learning policies through interaction with simulations. In Section 3 and Section 4, we describe the quantitative experiments and simulation results for the ecosystem under consideration, and we provide empirical results that validate the effectiveness of the RL agents in yielding reasonable outcomes. In Section 5, we analyze and discuss the qualitative behavior of the resulting agents. Finally, we conclude in Section 6, and sketch out some directions for future research.

The main contributions of this paper can be summarized as follows:

- First, we presented a simulation environment and learning agents that features predator-prey dynamics. In particular, we presented a novel procedure for co-evolution of predator-prey ecosystems by multi-agent reinforcement learning agents. More specifically, in the steps of the procedure for training predator and prey agents, we relied on a strategy, in which agents first compute approximate best response at each iteration, and then they try to strengthen their policies for responding well against the approximate best response. We also observed that the learned agent behaviors are somewhat ecologically plausible, in that they conform somewhat closely to results found in ecology research, e.g., the cycles in Lotka-Volterra equations [39,40].
- Second, we showcase some emergent features: RL-driven policies are qualitatively different from baseline random policies, yielding good solutions for both predators and preys. Moreover, RL-driven policies perform robustly with effective sustainability in the face of different initial conditions and/or environment sizes.
- Finally, we empirically show that throughout the scenarios resulting from co-evolution via multi-agent RL, predators found a reasonable means of survival, along with their preys, with a reduced risk of extinction.

## 2. Methods

The central concern of this paper is to examine the co-evolution of predator-prey ecosystems with an RL perspective, and this section presents a framework for studying the problem through numerical simulations with learning agents. In the following, we first describe the environment under consideration for interacting populations of predators and preys. The environment is a virtual ecosystem, in which predator and prey agents deal with partially observable states without explicit mutual communication. Next, we present reinforcement-learning-based reasoning about the emerging co-evolution in the predator-prey environment, which may be interpreted as similarity to what occurs in natural predator-prey ecosystems over a multitude of generations. Further related observations are to be provided later in the discussion section.

### 2.1. Environment Rules

**Figure 1:**
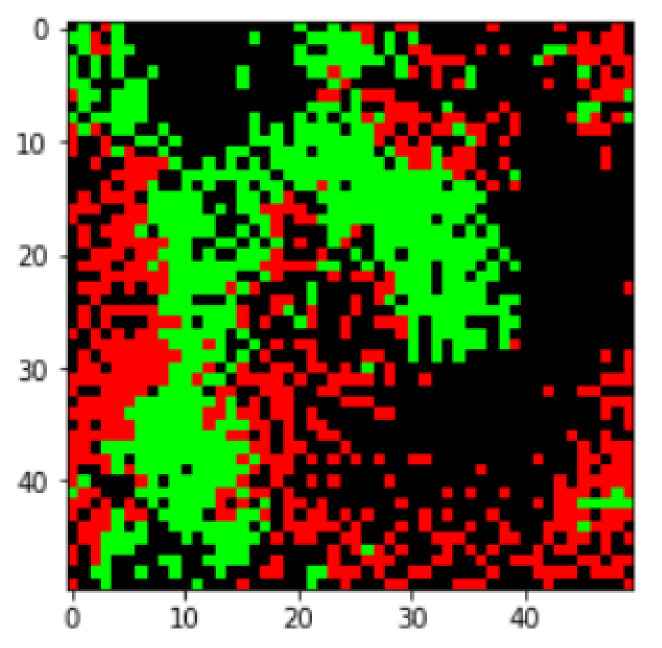
A visualization of the environment used for studying predator-prey ecosystems. The predators and prey agents are colored red and green, respectively.

In this subsection, we provide a detailed description of the environment for the virtual ecosystem under consideration, and its underlying dynamics for simulating predator-prey ecosystems. The environment is a grid world (see, e.g., Reference [36]) organized as a two-dimensional space in a quadrilateral form, with a lattice structure [41] consisting of N×N cells. We consider the N=50 scenario in the simulations, and other *N* scenarios will also be considered later in the discussion section. The lattice cells can be taken by predators or preys, or may remain empty. At the beginning of each episode, a certain number of cells are chosen randomly as the initial locations of agents. Spacial boundaries of the environment are assumed to be periodic (see, e.g., Reference [41]), in order to deal with a space similar to unbounded cases. Predators and preys are both agents capable of learning. This subsection focuses only on the simulation aspects of the agents (e.g., rules of the environment, and observations of agents), which will be necessary for performing Monte Carlo simulations for the environment. A visualization of the environment under consideration is shown in Figure 1.

- Dynamics of predators: A predator *X* can move to an adjacent cell which is not occupied by other agents. If the adjacent cell is already occupied by agents, two different rules are invoked, depending on the situation. If the adjacent cell is occupied by a prey organism, the predator can move and eat the prey in the cell while reproducing an offspring in the original cell with a success probability $b_{X}$. This reproduction follows the Bernoulli distribution with success probability $b_{X}$, which is denoted by $Bernoulli(b_{X})$. If the outcome of the Bernoulli reproduction is a failure, then the predator simply moves on and eats the prey without reproducing offspring. If the adjacent cell to which *X* intends to move is already occupied by another predator, predator *X* cannot move and, thus, remains in the original cell. In this environment, it is assumed that all predators have the same maximum permissible starvation level, $T_{X}$. Every time a step in the simulation passes without the predator eating, the predator’s starvation level increases, and when its starvation level reaches the maximum permissible level, $T_{X}$, then the predator is removed from the environment, yielding an empty cell.
- Dynamics of prey animals: A prey organism *Y* can move to an adjacent cell which is not occupied, and is able to reproduce an offspring with a success probability $b_{Y}$. If the outcome of the Bernoulli reproduction is a failure, then the prey moves to the next cell without reproducing offspring. When the adjacent cell to which *Y* intends to move is occupied by a predator, the prey *Y* will be eaten by the predator as a result of the movement. If the adjacent cell to which *Y* intends to move is already occupied by another prey organism, the prey animal *Y* cannot move, and remains in its original cell. In this environment, it is assumed that all the prey organisms have the same maximum age, $T_{Y}$. With the passage of each simulation step, the prey age increases, and, when its age reaches $T_{Y}$, the prey organism is removed from the environment, yielding an empty cell.
- Observations of agents: In this environment, each agent conducts its moves based on its perception of its neighborhood, which is the square scope with the size r×r. The agent spatial observations are padded as needed, when their observation window extends beyond the grid world. We consider the r=5 case in simulations here. Each cell in the neighborhood is either empty or occupied by an agent (i.e., a predator or prey); hence, the number of agents at each cell cannot exceed one.
- Rewards: The agents need to interact with an antagonistic definition of rewards, in that a desirable result for one class of agents is undesirable for the other class, for which we define the reward functions of agents as follows:
  (1) $$\begin{array}{ccc} Reward_{predator} & = & \left\{\begin{array}{c} +1,\;if\;a\;predator\;captures\;a\;prey\;animal\;as\;a\;result\;of\;its\;action\\ 0,\;otherwise \end{array}\right.\\ Reward_{prey} & = & \left\{\begin{array}{c} -1,\;if\;a\;prey\;animal\;is\;captured\;as\;a\;result\;of\;its\;action\\ 0,\;otherwise \end{array}\right. \end{array}$$

Finally, we assume that there is no explicit communication among agents in the framework.

### 2.2. Policies of Predators and Preys

**Figure 2:**
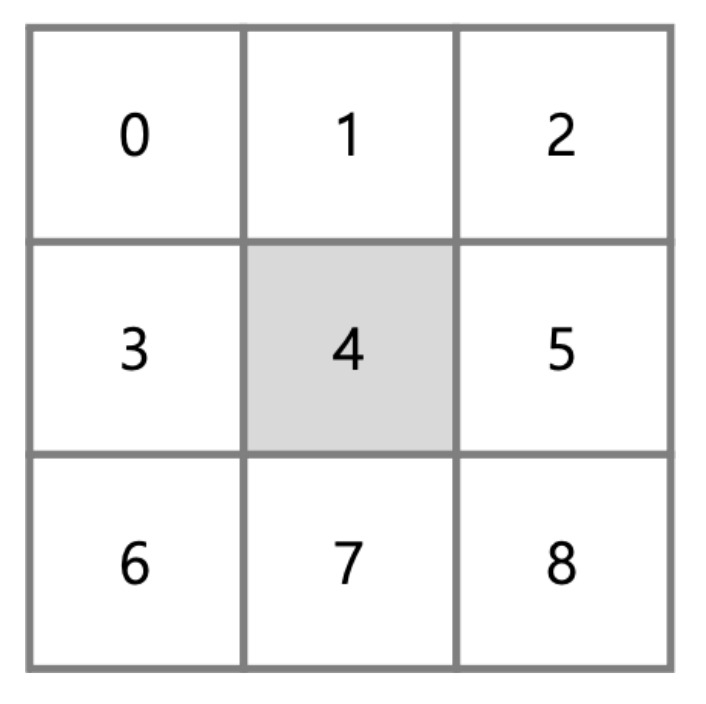
Discrete action space of the policy networks.

Agents navigate the environment in order to hunt or avoid being hunted. The action space of the agents includes nine actions for moving to an adjacent cell or remaining at the same location (Figure 2). As mentioned, agents are restricted from moving on top of cells occupied by other agents. An action consists of nine types of movement strategies shown in Figure 2. In our implementation, agents use the discrete action space (0: up-left, 1: up, 2: up-right, 3: left, 4: remain, 5: right, 6: down-left, 7: down, 8: down-right).

In this paper, we assume parameter sharing with decentralized execution [42] for each class of agents. Predators and prey organisms follow their own common policies $\pi_{\theta_{predator}}$ and $\pi_{\theta_{prey}}$, respectively. All predator (or prey) agents share the same parameters $\theta_{predator}$ (or $\theta_{prey}$) during training but condition their policies $\pi_{\theta_{predator}}\left(a_{i}|s_{i}\right)$ (or $\pi_{\theta_{prey}}\left(a_{i}|s_{i}\right)$) on agent-specific observations $s_{i}$. Note that the agent-specific observation, $s_{i}$, is the *r* by *r* restricted view of the grid-world’s true underlying global state s around the location of the agent *i*. In addition note that in this setting, if a predator (or prey) agent learns a useful new behavior in some area of the state space, then this may be available for other predator (or prey) agents by means of training with experience. In addition, note that predator (or prey) agent behaviors remain heterogeneous because they all have different observations. For simplicity, we express the parameters of predators and prey organisms collectively by θ. In addition, for convenience of presentation, we often write $\pi_{\theta }$ as π.

We make use of a deep neural network based on a multi-layer perceptron (MLP) with two hidden layers to model agent policies. The outcome features of the MLP are used to effectively control and find an approximation of the best response policy with off-policy reinforcement learning. The action of agent *i* at time *t* is sampled according to the conditional probability implemented by the network, i.e.,

(2) $$a_{i,t}∼\pi_{\theta }\left(a_{i,t}|s_{i,t}\right),i\in I,$$

where I is the collection of agents. The policy networks for predators and prey organisms are implemented by deep neural networks. The output of the policy network includes a probability distribution and the corresponding logit values over actions, and the input to the network consists of the agent-specific observations.

### 2.3. Multi-Agent RL-Based Learning of Agents

In this section, we are concerned with a solution to the problem of understanding some important interactions that arise in predator-prey ecosystems. For the solution procedure, we utilize the framework of modern multi-agents reinforcement learning (RL) in a model-free setting, in which prior knowledge of dynamics is not available for agents’ sequential-decision-making, and each class of agents (i.e., predators and prey) attempt to adaptively learn efficient policies by relying on their own previous experiences in the unknown environment. We present a multi-agent reinforcement learning procedure to perform the training of agents in a stable manner for the problem, including the learning of approximate best responses and entropy regularization.

As is well-known, the joint optimization problem posed by multi-agent reinforcement learning problems may cause challenging problems, such as non-stationarity and instability, during training [43,44]. Since multi-agent decision-making problems include the effect of other agents’ behaviors, encoded through agent policies, other agents’ behaviors essentially change the environment. In addition, in this predator-prey ecosystem, simultaneously training both predators and prey together may create an unstable learning landscape. To meet such challenges, we use a basic strategy [45,46,47] of iterating two stages for computing approximate equilibria in sequential adversarial games. In the first stage, we train prey agents, which is implemented by deep neural networks, for the purpose of computing approximate best responses, and with predator training paths detached from the backpropagation path. We also train predator agents similarly for their approximate best response with prey training paths detached from the backpropagation path. In the second stage, we perform data gathering and policy gradient (PG)-based update. In PG-based update, we perform a gradient ascent for agents, i.e., on the predator strategy to increase predators’ performance against approximately best responding prey organisms, and on the prey strategy to increase preys’ performance against approximately best responding predators. The use of a policy gradient in the step is due to the observation that in multi-agent problems, a policy gradient approach tends to perform better than other methods when using feed-forward neural architectures [42]. The established procedure based on the strategy is outlined in Table 1.

Note that since the policy of opponent agents is fixed, each stage deals with a standard reinforcement learning problem, in which agents iteratively explore and discover which behaviors are optimal for their objectives as defined via the reward function. As a result of the policy and behavior of opponent agents undergoing changes, the agent policy faces a non-stationary problem [43] and needs to learn adaptively in each iteration. We model the learning problem for agents in the predator-prey ecosystem as a discounted reward reinforcement learning problem [24] with states s∈S, actions a∈A, discount rate γ∈(0,1), and time steps t∈{0,1,⋯}, in which learning agents interact with a Markov decision process (MDP) environment [48], which is defined by the tuple (S,A,T,r,γ). In the MDP, the environment’s dynamics are characterized by state transition probabilities $T\left(s,a,s^{\prime }\right)\overset{▵}{=}Pr\left\{s_{t+1}=s^{\prime }|s_{t}=s,a_{t}=a\right\}$ and expected rewards $r\left(s,a\right)\overset{▵}{=}E\left[r_{t}|s_{t}=s,a_{t}=a\right]$. The learning agent takes actions following the policy described as a conditional probability $\pi \left(a|s\right)\overset{▵}{=}Pr\left\{a_{t}=a|s_{t}=s\right\}$, which is parametrized as $\pi_{\theta }$. The objective of the learning agent is to pursue a policy that can maximize the discounted expected return

(3) $$\rho \left(\pi_{\theta }\right)\overset{▵}{=}\left(1-\gamma \right)E_{\tau ∼P(\tau |\theta )}\left[\sum_{t=0}^{\infty }\gamma^{t}r\left(s_{t},a_{t}\right)\right],$$

where P(τ|θ) is the distribution over state-action trajectories $\tau =(s_{0},a_{0},s_{1},a_{1},\cdots )$ induced by the policy $\pi_{\theta }$ and transition probabilities $T(s,a,s^{\prime })$. The left-hand side of (3), $\rho (\pi_{\theta })$, is the objective of the optimization, and often referred to as the value of the policy. With the help of the related concepts, $Q^{\pi }$ and $d^{\pi }$, the value of the policy can be expressed in the frameworks of optimization. For a policy π, the state-action value function $Q^{\pi }\left(s,a\right)$ denotes the expectation of the future discounted reward sum of following π from the initial (s,a). In addition, $d^{\pi }\left(s,a\right)$ denotes how likely π is to visit (s,a) when interacting with environment. Note that, with the state-action value function

(4) $$Q^{\pi }\left(s,a\right)\overset{▵}{=}E[\sum_{t=0}^{\infty }\gamma^{t}r\left(s_{t},a_{t}\right)|s_{0}=s,a_{0}=a\left|\pi \right],$$

and the visit occupancy $d^{\pi }\left(s,a\right)\overset{▵}{=}\sum_{t=0}^{\infty }\gamma^{t}Pr\left\{s_{t}=s,a_{t}=a|\pi \right\}$, we can express the objective function of the solutions with the following primal and dual problems:

(5) $$\begin{array}{cc} \left(P\right) & \rho \left(\pi \right)=\min_{Q}\left(1-\gamma \right)E_{\mu_{0},\pi }\left[Q\left(s_{0},a_{0}\right)\right]\\ \; & s.t.\;Q\left(s,a\right)\geq r\left(s,a\right)+\gamma P_{\pi }Q\left(s,a\right),\forall s,a, \end{array}$$

(6) $$\begin{array}{cc} \left(D\right) & \rho \left(\pi \right)=\max_{d\geq 0}E_{d}\left[r\left(s,a\right)\right]\\ \; & s.t.\;d\left(s,a\right)=\left(1-\gamma \right)\mu_{0}\left(s\right)\pi \left(a|s\right)+\gamma P_{\pi }^{*}d\left(s,a\right),\forall s,a. \end{array}$$

Since both the linear nature and min-max form of the resultant Lagrangian functions may lead to numerical instability [49], the strategy of introducing additional regularization to the objective leads to a better curvature. By regularizing with the *f*-divergence $D_{f}\left(d∥d^{D}\right)$ [50] for solving the problem in a more stable manner, one can obtain the following modified dual version:

(7) $$\begin{array}{cc} \left(\tilde{D}\right) & \rho \left(\pi \right)-D_{f}\left(d∥d^{D}\right)=\max_{d\geq 0}E_{d}\left[r\left(s,a\right)\right]-D_{f}\left(d∥d^{D}\right)\\ \; & s.t.\;d\left(s,a\right)=\left(1-\gamma \right)\mu_{0}\left(s\right)\pi \left(a|s\right)+\gamma P_{\pi }^{*}d\left(s,a\right),\forall s,a. \end{array}$$

In addition, by taking the Fenchel-Rockafellar duality [49] of $\left(\tilde{D}\right)$, one can obtain the following modified primal version:

(8) $$\begin{array}{cc} \left(\tilde{P}\right) & \rho \left(\pi \right)=\min_{Q}\left(1-\gamma \right)E_{\mu_{0},\pi }\left[Q\left(s_{0},a_{0}\right)\right]\\ \; & +E_{\left(s,a\right)∼d^{D}}\left[f_{*}\left(r\left(s,a\right)+\gamma P_{\pi }Q\left(s,a\right)-Q\left(s,a\right)\right)\right],\forall s,a. \end{array}$$

Note that real meaning about the use of (8) is the advantages coming from the convexity in unconstrained formulation. In this paper, we deal with the nested problems in Table 1 by solving ($\tilde{P}$) iteratively for finding an approximate best response policy. In the process of solving ($\tilde{P}$), we follow the strategy of AlgaeDICE [51], in which the θ of the actor $\pi_{\theta }$ is updated via a policy gradient, and the parameters related with the *Q* and *d* are fit by optimizing the objective function of $\left(\tilde{P}\right)$. In regularization with *f*-divergence for the modified problems $\left(\tilde{P}\right)$ and $\left(\tilde{D}\right)$, we used $f\left(x\right)=x^{2}/2$. As mentioned, agent policies are implemented within the structure of deep neural networks, the inputs and outputs of which are for observations and actions, and their weights are trained as shown in the procedure shown in Table 1. Finally, in the process of training the neural networks for agents, we use entropy regularization, which adds the policy’s entropy [52,53] as an additional weighted term in the policy gradient objective. The use of the entropy regularization term, which is defined as

(9) $$Entropy\left(\pi_{\theta }\right)=-E_{a∼\pi_{\theta }\left(\cdot |s\right)}\left[\log\pi_{\theta }\left(a|s\right)\right],$$

promotes more effective exploration by agents when used with policy gradient (PG).

**Table 1:** An established procedure for co-evolution of predator-prey ecosystems by reinforcement learning (RL) agents.

**Given:** - Sampling horizon *h* - Off-policy single agent RL algorithm A (AlgaeDICE [51]) - Stopping criterion C (e.g., maximum number of iterations, flag indicating that the performance indices have not improved)
**Goal:** To find trained results for agent policy networks
**Procedure:** Initialize policy networks Reset experience buffer D Reset episode For each sampling horizon, do the following: Compute an approximate best response for each agent policy via RL algorithm A Data collection: For each environment step, do - Agents take actions based on their policies; $a_{i,t}∼\pi \left(a_{i,t}|s_{i,t}\right),\forall i\in I$ - Environment changes via environment rule; $s_{t+1}∼EnvRule\left(s_{t+1}|s_{t},a_{t}\right)$, where $a_{t}=\left(a_{1,t},\cdots ,a_{|I|,t}\right)$ - Collect state transition data Update experience buffer D by adding the collected data, and conduct gradient update step for policy networks Termination check with criterion C, and if not satisfactory, go to step 3

## 3. Quantitative Experiments

In this section, we perform simulations to empirically validate the proposed learning mechanism, in which reasonable behaviors of agents emerge as a result of trained policies. We provide empirical results that validate the effectiveness of the trained policies in finding stable and ecologically plausible outcomes.

At the start of each episode, a given number of agents are randomly spawned in the cells. The state of the environment is represented as a H×W×C tensor, where *H* and *W* are the size of the grid world, *C* is the number of unique entities that may occupy a cell, and the value of a given element indicates that a particular entity occupies the associated location.

We use AlgaeDICE [51] and the AdamW optimizer [54] to compute policy gradients. Samples were collected using a sampling horizon of h=70 time steps between policy update iterations. Our experiments were conducted on a computing environment with a CPU (Intel i5-7287U) and GPU (Nvidia Titan XP), and performing the training of a single iteration for an episode took 14.7 s on average. In the process of training the neural networks for agents, we used PyTorch [55], which provided a convenient end-to-end framework with familiar building blocks. In addition, for the data plotting and visualization, we used the plotting package Matplotlib [56].

We evaluated our method via simulations. The environment of simulations is a two-dimensional grid world with both height and width of N=50. At the beginning of the simulation, $n_{X}=100$ predator agents and $n_{Y}=500$ prey agents are randomly located on the grid for the initial state. As time step increases occur, agents change their positions according to their policies and the environment rule. The predator and prey reproduce offspring with probabilities of $b_{X}=0.2$ and $b_{Y}=0.6$, respectively. The predator’s starvation level and prey age are initialized at 0. In addition, the maximum starvation level of predators is $T_{X}=15$, and the maximum age of prey animals is $T_{Y}=30$. The parameter set used for the simulations is outlined in Table 2.

## 4. Simulation Results

In this section, we report the simulation results, and provide a performance comparison with some baseline cases. We simulate episodes for training the model, and in the simulations, agent locations are updated sequentially following the environment rule. Each episode is terminated when its time step reaches a fixed maximum length, or when one of the species becomes extinct.

**Figure 3:**
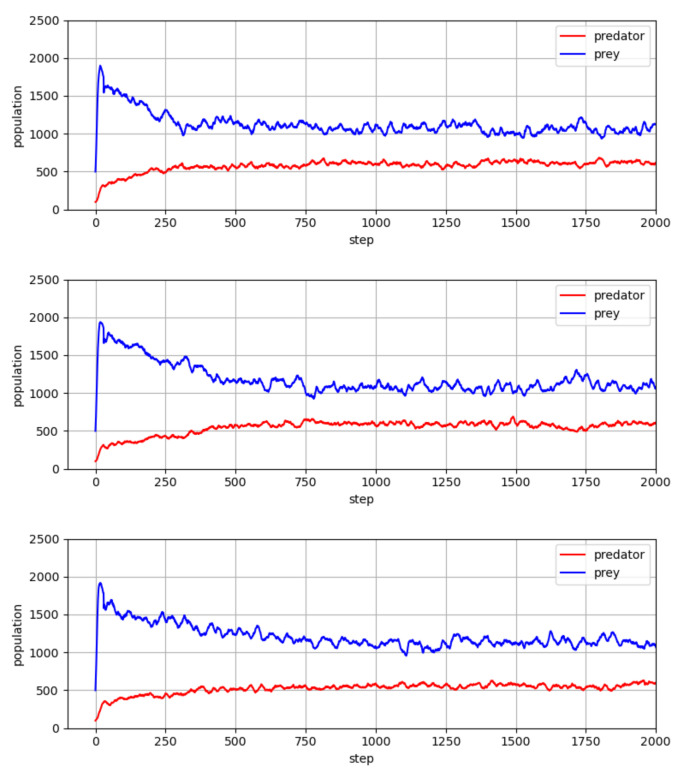
Population values of predator and prey, which went through the co-evolution process of Table 1.

In Figure 3, we report our main results with three different random seeds, in which the predator and prey policies went through the co-evolution process of Table 1. We empirically found that the maximum number of iterations is a reasonable stopping criterion for the quantitative experiments for the present paper. More specifically, we performed all simulations with a couple of tens of iterations for steps 3, 4, and 5 in the co-evolution process, which we found to be sufficient for both predators and preys to converge to relatively stable policies. As shown in the figure, there are some patterns in the predator and prey populations. In both species, the population size fluctuates around a certain value, and this fluctuation continues while maintaining a certain level of gap. This oscillating pattern may be interpreted as follows: with the population growth of predators, more prey animals would be captured, and as a result of these captures, the prey population would be reduced. Then a reduction of the predator population would follow due to the reduction in the prey population, and this cycle seems to occur continuously.

**Figure 4:**
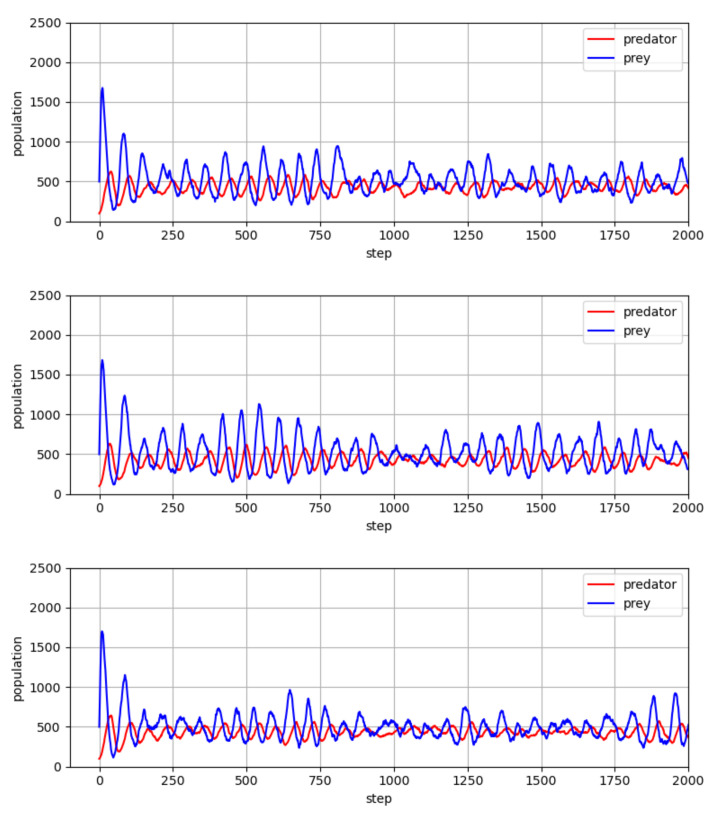
Population values of predator and prey, which result from random policies.

For comparison, we considered cases in which the agents interact with random policies. Figure 4 shows the changing population of predators and prey with time. From Figure 4, one can see that the random policies can induce fluctuation in the population of predators and prey, which bears some resemblance with the results of Figure 3. However, there are two critical differences in these fluctuations brought about by random policies: First, the population dominance of prey over predators is not obvious in the population fluctuations; and, second, the population values of predators and preys exhibit large fluctuations around low values, which may lead to a risk of their extinction.

A comparison of the simulation results in Figure 3 and Figure 4 indicates that the co-evolution process of Table 1 brings some positive effects for the predator-prey ecological system, in that the populations of both species increased with a desirable ecological dominance and became stable.

**Figure 5:**
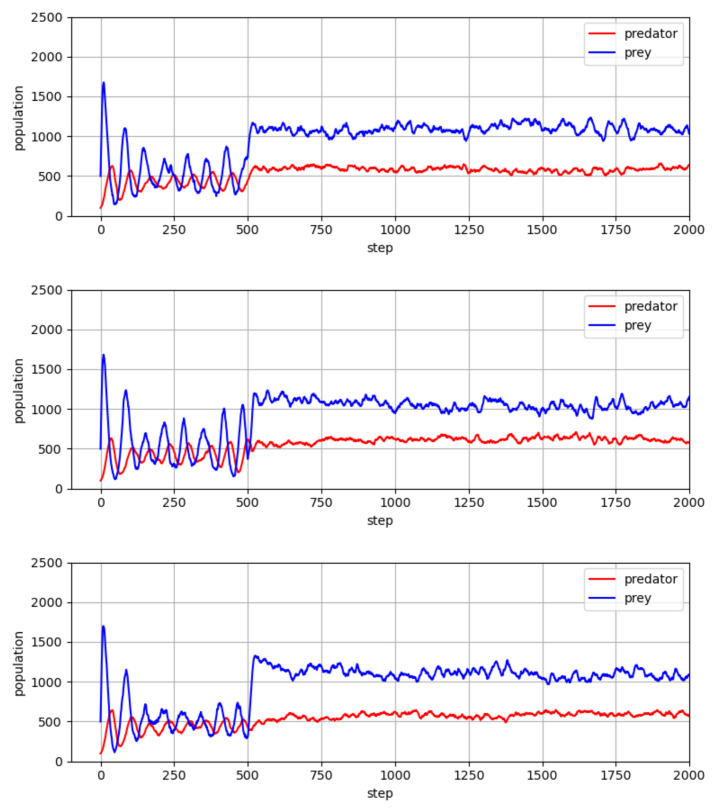
Population of predators and prey, which show a transition from random to trained policies at t=500.

For the final experimental issue of this section, we observe whether the trained policies have some robustness when they are started from random. More precisely, predators and prey take random actions during the first 500 steps. After 500 steps, predators and prey move according the policies on which they are trained by means of the co-evolution process as in Table 1. Simulation results of Figure 5 show that during the initial 500 steps, all three cases exhibit high fluctuation with the risk of extinction. One can see from the figure that the transition from initial high fluctuation to a stable balance occurs successfully around the change point at t=500, and the magnitude of fluctuations after 500 steps becomes smaller. In addition, the average population values of predator and prey increased compared to their values in the initial 500 steps, leading to a significant reduction of extinction risk. Some more points concerning the robustness of the trained agent policies will be discussed in the next section.

## 5. Discussion

In this paper, we investigated the use of multi-agent reinforcement learning for characterizing the dynamic nature of predator-prey ecosystems. Recently, reinforcement learning methods have achieved impressive advances in areas, such as games and robotics. Reinforcement learning agents focus on building strategies that lead to taking actions in an environment in order to maximize expected rewards. The key idea behind our characterization is to frame the co-evolution of predators and preys in an ecosystem as enabling learning agents to try to optimize for equilibrium policies in a manner appropriate for multi-agent reinforcement learning. As mentioned in the section of introduction, there have been important MARL efforts which are related with the present paper. These works may look somewhat similar, but details, such as what type setting (e.g., cooperative, competitive, and mixed) the problem handles, what are the assumptions for the agents, and how training proceeds, are different. For example, one of the related MARL efforts is the stable emergent policy approach called STEP of Reference [31]. The present paper and STEP share some similar features because both are multi-agent reinforcement learning efforts, and the trained polices are executed in a decentralized fashion in the sense that each agent takes action conditioned on its own observation, whereas the focus of STEP is somewhat different from our works in that they deal with cooperative tasks and rely on the planning enabled by a simulator. We believe that our final results are distinctive in explaining the considered ecological issues, and working well when planning is not available.

**Figure 6:**
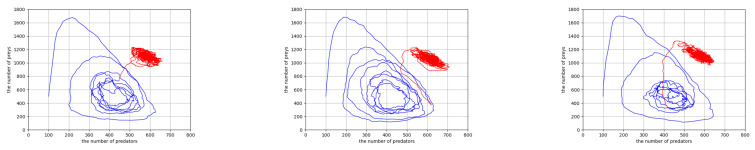
Emergent behaviors similar to cycles in the Lotka-Volterra equation. Initial and later locations are colored blue and red, respectively.

The problem under consideration may be described as a decentralized version of a partially observable multi-agent Markov game, and its solutions may be sought by finding Nash equilibria. A set of optimal policies form a Nash equilibrium as long as no agent wants to unilaterally deviate from its own policy. Finding exact equilibria for the ecosystems considered might be intractable and is beyond the scope of this paper. Nevertheless, we observed that a multi-agent RL-based procedure can yield reasonable co-evolution, along with emergent fluctuations somewhat similar to cycles in the Lotka-Volterra equation [39,40]. In Figure 6, the relation between the population of predators and prey is represented by a periodic pattern of fluctuations. As in Figure 5, simulations for Figure 6 began with random policies in their initial 500 steps. Then both species moved according to the policies by which they were trained via co-evolution in Table 1, which results in average population values of predators and prey increasing over the values of the initial period. During the initial 500 steps with random policies, the radius of the circle representing the magnitude of population variations of predators and prey is relatively large. After 500 steps, the cycles assume a reduced size, and their movements around the centers become more sustainable, in that they are maintained with a reduced risk of extinction. It was found that the mid-point of the circle moved to the top right after 500 steps.

**Figure 7:**
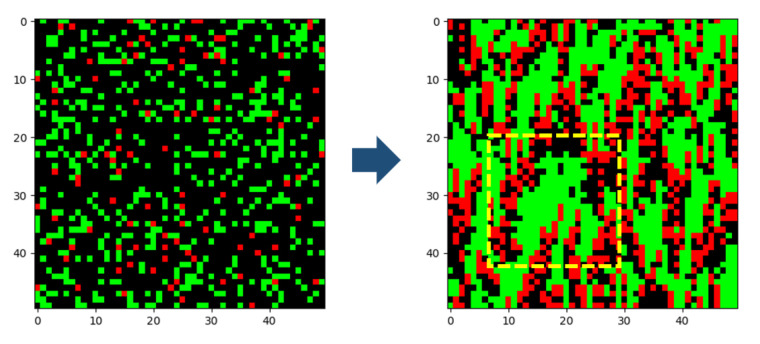
**Left**: Initial random location of predators and prey. **Right**: Emergence of swarming among predators and prey.

As an additional pattern observed from the numerical experiments, we can see emergence of swarming in Figure 7. Self-organization in the form of swarming is a well-studied process in the field of population biology. This concerns the emergence of globally ordered population dynamics in space and time, realized from collective interactions between agents without any explicit external intervention. Figure 7 shows the emergence of swarming in the movements of the trained predators and prey organisms. The left columns of the figure shows the initial random locations of species, while the right columns show locations when their time steps reach t=2000. From this figure, one can see that prey organisms exhibit a tendency of swarming for better survival from attempted capture by predators, while predators tend to swarm for convenience in hunting with fewer movements. Note that swarming here is simply a consequence of agents learning to maximize their own individual rewards, without direct intervention from the environment.

**Figure 8:**
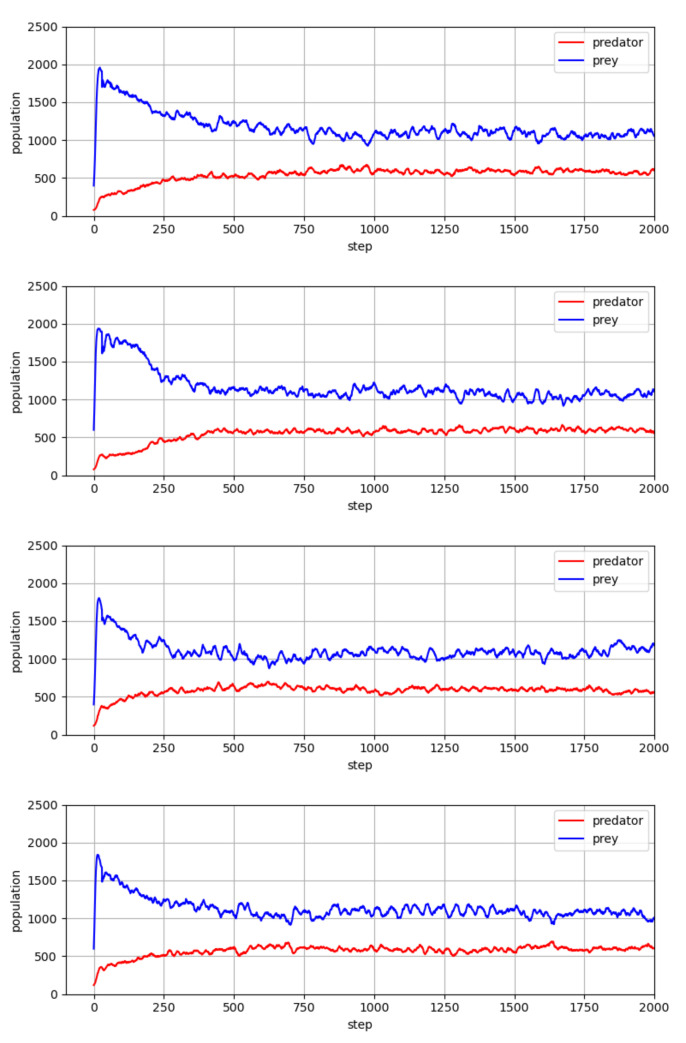
Population values of predator and prey, which went through the co-evolution process of Table 1. Simulation results show that learned agent policies can be effective without additional parameter tuning for different initial conditions (Initial numbers of predators and preys, $n_{X}$ and $n_{Y}$, are changed by ±20%.).

**Figure 9:**
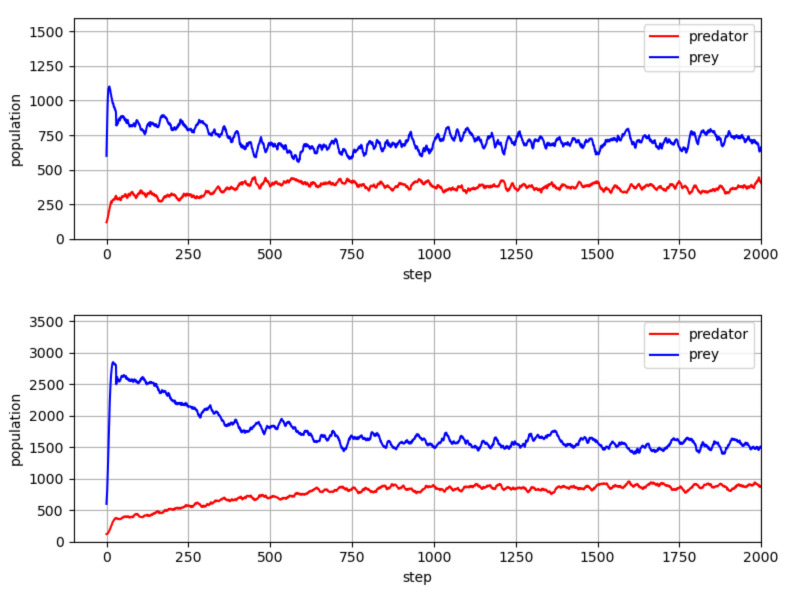
Population values of predator and prey, which went through the co-evolution process of Table 1. Simulation results show that learned agent policies can be effective without additional parameter tuning for different environment sizes (N=40 and N=60 cases).

We also investigated whether the learned agent policies can be effective without additional parameter tuning for different initial conditions (e.g., Figure 8) or in environments with different sizes (e.g., Figure 9). The investigations show that the trained agent policies resulting from the procedure of Table 1 work effectively for different initial conditions, and scales well for to a smaller or larger environment size. The positive results in this investigation suggest that by learning with principles of multi-agent reinforcement learning, agents can achieve robust performance against deviation from a nominal environment.

**Figure 10:**
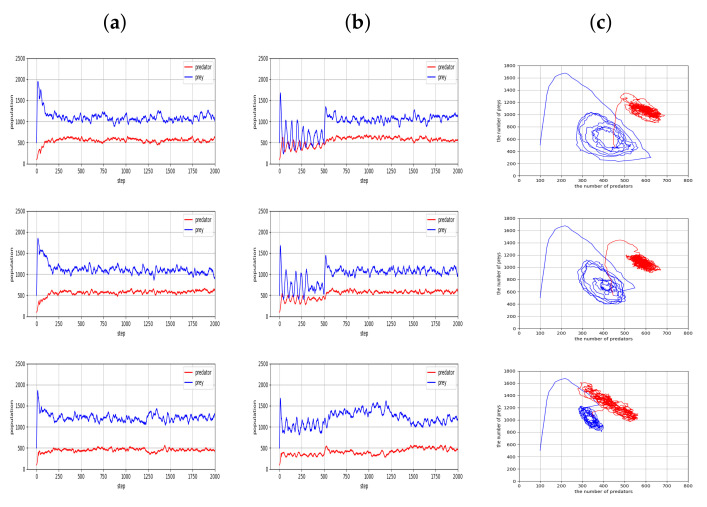
Simulation results for the cases trained with the maximum age of predator set as 40 (**a**), 30 (**b**), and 20 (**c**).

For enhanced realism in the model, we also conducted simulations for cases with predator age considered. As shown in Figure 10, it turns out that the simulation results remain almost the same when the maximum age of predator is older than that of prey, as is common in real-world predator-prey situations. We also observed that when the predator age is shorter than the prey age, the results become somewhat different. Detailed analysis of reasons for this difference is one of the topics to be addressed in future studies.

For performance comparison with an existing approach, we considered an approach based on deep Q-learning (DQN) [25]. As is well-known, using deep neural networks along with Q-learning [24] today is an important method for reinforcement learning, and has been utilized extensively for problems involving sequential decision-making. The problem of formalizing the co-evolution of predators and preys as a deep Q-learning-based reinforcement learning was recently introduced in Wang et al. [36]. In addition, their subsequent work [37] adopted neural networks and presented a similar deep Q-learning-based evolution mechanism for predator-prey ecosystems, with discretized features for states. These deep Q-learning-based works rely on the update mechanism of *Q* in the following form:

$$Q\left(s,a\right)\leftarrow \left(1-\alpha \right)Q\left(s,a\right)+\alpha [reward+\gamma \max_{a_{next}\in A}Q\left(s_{next},a_{next}\right)].$$

**Figure 11:**
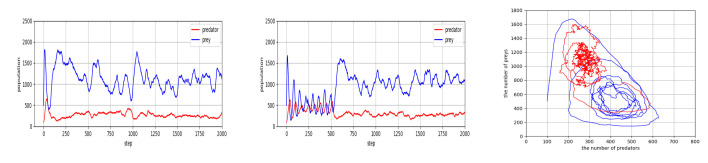
Simulation results for the cases trained with Deep Q-Networks (DQN)-based approach.

The simulation results of Reference [36] show that when the predators and preys co-evolved, predators and preys updated their networks according to each other’s behavior, which led to the reduced oscillations of the ecosystem. We compared the results of the proposed method to those of the DQN-based approach [36] with ϵ=0.05 for exploration, and learning rates for the predator and the prey policy networks set as $10^{-5}$ and $10^{-3}$, respectively. Figure 11 shows the corresponding results obtained by the DQN-based approach. One can observe that the trajectories in the center of Figure 11 looks somewhat similar to the corresponding case reported in Figure 3 of Reference [36]. A comparison shows that our results are better, in that the resulting co-evolution of predators and prey brings about more stable ecological systems.

Finally, simulation studies, like ours, may naturally have some limitations. For example, they are not based on real world data on agents’ behaviors and interactions, and consider a relatively small environment in order to avoid enormous computational loads. If demonstrations of predator and prey agents with successful hunting and/or survival skills are provided, along with relevant information, then, in principle, their rewards could be estimated via so-called multi-agent adversarial inverse reinforcement learning [57] and used with the procedure. Such demonstration data are currently not available to the best of authors’ knowledge, and further study along these lines is a topic that warrants future research. Future simulations could further improve on this by making use of large-scale reinforcement learning methods with explicit targets conforming to real-world observations. For improved follow-up research, one may also consider this problem on a larger scale, e.g., so that co-evolution in digital and/or business ecosystems [58] can be included.

## 6. Concluding Remarks

In this paper, we use a modern deep reinforcement learning (RL) approach to explore a new avenue for understanding key population dynamics of predator-prey ecosystems. Reinforcement learning methods have achieved impressive results, and reinforcement learning agents generally focus on building strategies that lead to agents taking actions in an environment in order to maximize expected reward. In this paper, we frame the co-evolution of predators and preys in an ecosystem as building learning agents that optimize for equilibrium policies in a manner appropriate for multi-agent reinforcement learning. This novel approach leads to helpful insights on these types of complex problems.

Our simulation results show that throughout the scenarios with reinforcement learning agents, predators can find a reasonable level of sustainability, along with their prey, and co-evolution of predators and preys brings about stable ecological systems. In addition, we found that training with multi-agent and model-free reinforcement learning can yield agents with ecologically plausible behaviors, such as population fluctuations, around some constant values, and the emergence of swarming. We believe that, with a combination of a variety of ecosystems and modern reinforcement learning methods, one can find a wide range of important results. In future works, we would like to explore these combination in related fields, where agents play more general roles. We also plan to investigate the robustness of our method for environments with more general features.

## Figures and Tables

**Table 2:** Parameter set used in simulations.

Notation & Value	Meaning
$b_{X}=0.2$	Reproduction Probability of Predator
$b_{Y}=0.6$	Reproduction Probability of Prey
$T_{X}=15$	Maximum Starvation Level of Predator
$T_{Y}=30$	Maximum Age of Prey
$n_{X}=100$	Initial Number of Predators
$n_{Y}=500$	Initial Number of Preys

## Data Availability

Not applicable.
